# *Agrobacterium tumefaciens*-mediated transformation of *Aspergillus aculeatus *for insertional mutagenesis

**DOI:** 10.1186/2191-0855-1-46

**Published:** 2011-12-14

**Authors:** Emi Kunitake, Shuji Tani, Jun-ichi Sumitani, Takashi Kawaguchi

**Affiliations:** 1 Graduate School of Life and Environmental Sciences, Osaka Prefecture University, 1-1 Gakuen-cho, Naka-ku, Sakai, Osaka 599-8531, Japan

**Keywords:** TAIL-PCR, gene tagging, insertional mutagenesis

## Abstract

*Agrobacterium tumefaciens*-mediated transformation (AMT) was applied to *Aspergillus aculeatus*. Transformants carrying the T-DNA from a binary vector pBIG2RHPH2 were sufficiently mitotically stable to allow functional genomic analyses. The AMT technique was optimized by altering the concentration of acetosyringone, the ratio and concentration of *A. tumefaciens *and *A. aculeatus *cells, the duration of co-cultivation, and the status of *A. aculeatus *cells when using conidia, protoplasts, or germlings. On average, 30 transformants per 10^4 ^conidia or 217 transformants per 10^7 ^conidia were obtained under the optimized conditions when *A. tumefaciens *co-cultured with fungi using solid or liquid induction media (IM). Although the transformation frequency in liquid IM was 100-fold lower than that on solid IM, the AMT method using liquid IM is better suited for high-throughput insertional mutagenesis because the transformants can be isolated on fewer selection media plates by concentrating the transformed germlings. The production of two albino *A. aculeatus *mutants by AMT confirmed that the inserted T-DNA disrupted the polyketide synthase gene *AapksP*, which is involved in pigment production. Considering the efficiency of AMT and the correlation between the phenotypes and genotypes of the transformants, the established AMT technique offers a highly efficient means for characterizing the gene function in *A. aculeatus*.

## Introduction

The imperfect fungus *Aspergillus aculeatus *no. F-50 [NBRC 108796], which was isolated from soil in our laboratory, forms black-pigmented asexual spores similar to those of *Aspergillus niger*. This *A. aculeatus *strain produces cellulases and hemicellulases that are applicable for synergistic pulp hydrolysis in combination with cellulases from *Trichoderma reesei *(Murao et al. 1979). Another feature of *A. aculeatus *is its ability to secrete endogenous proteins in high quantities; *A. aculeatus *expresses its own β-mannosidase at levels 9 times greater than those of *A. oryzae*, which is one of the most widely used hosts for protein production (Kanamasa et al. 2007). Therefore, we aimed to genetically modify *A. aculeatus *to create a high-quality host for the production of autologous cellulases and hemicellulases, and thereby facilitate the production of effective enzymes for the saccharification of unutilized cellulosic biomass and its subsequent bioconversion. To achieve this goal, a method to increase the amount of secreted enzymes is necessary. Although it is important to understand the molecular mechanisms underlying the effective secretion of endogenous enzymes and the associated gene regulation mechanisms, these mechanisms remain unclear (Ooi et al. 1999; Takada et al. 1998 and 2002). Thus, there is an increasing need to establish methods for functional genetic analyses in *A. aculeatus*.

Random insertional mutagenesis is an efficient forward genetic technique for identifying the cellular roles of genes. One valuable method entails transferring a known gene into the recipient genome at random, as analyses of the phenotypes resulting from gene inactivation or modification can provide insight into the function of the affected genes. Transposon-mediated directed mutations and restriction-enzyme-mediated integrations (REMI) have long been applied for random insertional mutagenesis in fungal species (Braumann et al. 2007; Brown et al. 1998; Daboussi 1996; Linnemannstöns et al. 1999). However, both methods tend to multiply the transposable elements or transfer multiple copies of inserted plasmids into the recipient genome. These phenomena are disadvantageous when performing insertional mutagenesis in filamentous fungi such as *A. aculeatus*, for which a feasible genetic segregation analysis is unavailable. Recently, there has been a trend toward adopting *Agrobacterium tumefaciens-*mediated transformation (AMT) for insertional mutagenesis; this method has been widely used as a genetic engineering technique for plant cells (Feldmann 1991; Koncz et al. 1992) and more recently adapted to fungi including *Magnaporthe oryzae *(Betts et al. 2007; Meng et al. 2007), *Fusarium oxysporum *(Mullins et al. 2001), *Colletotrichum lagenarium *(Tsuji et al. 2003), *Cryptococcus neoformans *(Idnurm et al. 2004), *Aspergillus fumigatus *(Sugui et al. 2005), and *Aspergillus awamori *(de Groot et al. 1998). This transformation technique utilizes the ability of *A. tumefaciens *to transfer DNA (so-called T-DNA, which is located between two direct repeats, i.e., the left and right borders) to its host cells in the presence of a phenolic compound such as acetosyringone. The T-DNA is transferred as a single-stranded DNA into recipient cells by the Type IV secretion system (Backert and Meyer 2006; Christie 2001) and predominantly integrated as a single copy into the transformant genome (Betts et al. 2007; Michielse et al. 2005b; Tsuji et al. 2003).

Although it has been previously demonstrated that *A. tumefaciens *is capable of transforming various fungi including the Ascomycetes, the transformation conditions must be optimized because the transformation frequencies vary among fungal species and strains. To establish an efficient AMT method for high-throughput insertional mutagenesis in *A. aculeatus*, we optimized the AMT conditions to effectively isolate transformants harboring single-copy T-DNA insertions at random loci. We also demonstrated that the established AMT method is applicable for functional genetic analyses.

## Materials and methods

### Strains and plasmids

*A. tumefaciens *C58C1 and the binary vector pBIG2RHPH2, which carries a hygromycin B-resistant gene between the left and right T-DNA borders, were kindly provided by Dr. Tsuji (Tsuji et al. 2003). *A. aculeatus *strains were propagated at 30°C in minimal media (MM) supplemented appropriately, unless stated otherwise (Adachi et al. 2009). Conidia of transformants were purified by repeating mono-spore isolation twice on MM plates to obtain the conidia of homokaryons.

### Cloning and expression of *AapksP*

The polyketide synthase gene *AapksP *along with the regions 1,041-bp upstream and 567-bp downstream of the open reading frame was amplified by PCR with PrimeSTAR HS DNA polymerase (TaKaRa, Japan) and the primers pks-F_Nhe and pks-R_Nhe (Table 1) using *A. aculeatus *genomic DNA as a template. PCR condition is as described in manufacture's instruction except for setting annealing temperatures and PCR cycles as 65°C and 30 cycles. The amplified DNA fragments were sequenced, digested with *Nhe *I, and ligated into pAUR325 (TaKaRa, Japan) to yield pAUR-PksP. The transformation of *A. aculeatus *was performed by the protoplast method (Adachi et al. 2009) using the circular plasmids pAUR325 and pAUR-PksP. Transformants were selected on 3.5 μg/ml Aureobasidin A.

**Table 1:** Primers used in this study

Name	Sequence (5' to 3')	
HS-1com1	TGCTCCATACAAGCCAACC	
HAS-2com	ATCATCTGCTGCTTGGTGC	
AD-1	NGTCGASWGANAWGAA	
AD-2	GTNCGASWCANAWGTT	
AD-3	WGTGNAGWANCANAGA	
HS-1	GGCCGTGGTTGGCTTGTATGGAGCAGCAGA	436 bp from nick site in RB^a^
HS-2	TGGTCTTGACCAACTCTATCAGAGCTT	336 bp from nick site in RB
HS-3	GGACCGATGGCTGTGTAGAAGTA	193 bp from nick site in RB
HS-4	CTCGCCGATAGTGGAAACC	170 bp from nick site in RB, for sequencing
HAS-2	GCACCAAGCAGCAGATGAT	373 bp from nick site in LB^b^
HAS-3	AATAATGTCCTCGTTCCTGTCTGCTAATAA	354 bp from nick site in LB
HAS-4	CCGCCTGGACGACTAAAC	225 bp from nick site in LB
HAS-5	GACCTCCACTAGCTCCAGCC	187 bp from nick site in LB, for sequencing
pks-F_Nhe	taggctagcGTAAGCTCACCGTCAAGGCA	
pks-R_Nhe	ctggctagcAGATCCTAGAGACCCGGGAC	

^a^RB, right border; ^b^LB, left border

### *Agrobacterium tumefaciens*-mediated transformation (AMT)

AMT was performed as described in Tsuji et al. (2003) with minor modifications. *A. tumefaciens *C58C1 harboring pBIG2RHPH2 was grown in liquid LB medium supplemented with 30 μg/ml of kanamycin and 100 μg/ml of rifampicin at 28°C for 18 hours. The culture was diluted to an optical density at 660 nm (OD_660_) of 0.15 in 100 ml of induction medium (IM) with 200 μM acetosyringone (AS), 30 μg/ml of kanamycin, and 100 μg/ml rifampicin. The cells were grown at 24°C until the OD_660 _reached 0.2-0.8. The average numbers of *A. tumefaciens *cells in 100 μl of culture medium at OD_660 _= 0.2, 0.4, 0.6, 0.8, and 1.0 were calculated as 2.5 × 10^7^, 5 × 10^7^, 7.5 × 10^7^, 1 × 10^8^, and 1.25 × 10^8 ^cells, respectively, using a colony-counting method. In the co-cultivation on solid IM, a mixture of 100 μl of *A. tumefaciens *suspension and 10^4 ^*A. aculeatus *conidia was spread onto filter paper (hardened, low-ash grade 50; Whatman, Maidstone, UK) on IM containing 200 μM acetosyringone (AS). After co-cultivation for 24-72 h at 24°C, the filter paper was transferred to the selection medium (SM; MM containing 100 μg/ml of hygromycin B and 100 μg/ml of cefotaxime). When co-cultivation was performed in liquid IM, *A. tumefaciens *was cultured to OD_660 _= 0.4, harvested by centrifugation, and co-cultivated with 10^7 ^of *A. aculeatus *conidia in liquid IM containing 200 μM AS. After shaking at 120 rpm for 16-96 hours at 24°C, the germlings were harvested and incubated on SM.

### Molecular analyses of transformants

Conidia from the transformants were grown in MM containing 100 μg/ml of hygromycin B at 30°C for 50 hours on a shaker (170 rpm). Genomic DNA was isolated as described in Adachi et al. (2009) from mycelia and was digested with *Eco*R I and *Sal *I or *Xba *I and *Hin*d III. The *Eco*R I and *Xba *I recognition sites are located within the T-DNA region at positions 124 and 81 nt from the left and right border nick sites, respectively. The digestion of genomic DNA with *Eco*R I or *Xba *I in combination with *Sal *I or *Hin*d III, for which there are no recognition sites on pBIG2RHPH2, yields relatively shorter fragments and thus helps to distinguish the fragment size. Hybridization was performed as described in Adachi et al. (2009) using an 880-bp fragment amplified with *hph*-specific primers (HS-1com1 and HAS-2com) as a DNA probe (Table 1).

A thermal asymmetric interlaced polymerase chain reaction (TAIL-PCR) was performed to obtain DNA sequences flanking the T-DNA insertions in the fungal transformants, following the methods described in Liu et al. (1995) and Sessions et al. (2002) with minor modifications, as summarized in Table 2. The T-DNA specific (left border, HAS-2-4; right border, HS-1-3) and arbitrary degenerate primers (AD1-3) are described in Table 1. The final concentrations of the T-DNA-specific primers were adjusted to 0.4 μM and those of the AD primers were 3-4 μM (depending on the degree of degeneracy) in the primary reaction and 2 μM in the secondary and tertiary reactions. The amplified tertiary PCR products were subjected to agarose gel electrophoresis and sequence analysis. TAIL-PCR was also performed with a recipient genome digested with *Bgl *II, *Eco*R I or *Xba *I. *Bgl *II sites are located outside the T-DNA region at positions 511 and 133 nt from the left and right border nick sites, respectively. Thus, digestion with these restriction enzymes produces T-DNA fragments carrying either side of the flanking sequence tag even when the T-DNA, with or without the vector backbone, is integrated into a recipient genome as concatemeric bands.

**Table 2:** Thermal settings for TAIL-PCR

Reaction and cycle	Thermal settings
Primary	
1	93°C, 1 min.; 95°C, 1 min.
5	98°C, 30 sec.; 62°C, 15 sec.; 72°C, 3 min.
	98°C, 30 sec.; 25°C, 3 min.;
1	ramping to 72°C, over 3 min.; 72°C, 3 min.
	98°C, 10 sec.; 68°C, 15 sec.; 72°C, 3 min.;
15	98°C, 10 sec.; 68°C, 15 sec.; 72°C, 3 min.;
	98°C, 10 sec.; 44°C, 15 sec.; 72°C, 3 min.
1	72°C, 5 min.
Secondary	
1	93°C, 2 min.
	98°C, 10 sec.; 64°C, 15 sec.; 72°C, 3 min.;
12	98°C, 10 sec.; 64°C, 15 sec.; 72°C, 3 min.;
	98°C, 10 sec.; 44°C, 15 sec.; 72°C, 3 min.
1	72°C, 5 min.
Tertiary	
1	93°C, 2 min.
	98°C, 10 sec.; 68°C, 15 sec.; 72°C, 3 min.;
12	98°C, 10 sec.; 68°C, 15 sec.; 72°C, 3 min.;
	98°C, 10 sec.; 44°C, 15 sec.; 72°C, 3 min.
1	72°C, 5 min.

Inverse PCR was also applied to rescue the flanking sequences. Genomic DNA from each transformant was digested with *Nco *I, *Nde *I (both located in the middle of the T-DNA), *Eco*R I, or both *Xba *I and *Spe *I and used as a template for inverse PCR. *Spe *I was used to increase the possibility of obtaining fragments flanking the T-DNA because there are no *Spe *I recognition sites inside of the T-DNA, and this enzyme yields cohesive ends that are complementary with those produced by *Xba *I. Using genomic DNA digested with *Nco *I or *Nde *I as templates, the flanking sequences adjacent to the left and right borders were amplified with the primer sets HAS-4 and HAS-2com or HS-3 and HS-1com1, respectively. When genomic DNA digested with *Eco*R I or *Xba *I/*Spe *I was used as the template, the flanks of both sides of the borders were amplified with the primer sets HAS-4 and HS-3, respectively. The amplified DNA fragments were sequenced with the primer sets HS-4 and HAS-5.

### Mitotic stability

Nine randomly selected transformants were cultured on MM in the absence of hygromycin B for 5 generations. Approximately 100 conidia derived from each 5th generation were spread on MM with or without 100 μg/ml of hygromycin B.

## Results

### *A. tumefaciens*-mediated transformation (AMT) of *A. aculeatus *no. F-50 on solid IM

To determine whether or not AMT is applicable for *A. aculeatus *transformation, we first co-cultivated 1 × 10^4^, 10^5^, or 10^6 ^wild-type *A. aculeatus *conidia and an *A. tumefaciens *culture at OD_660 _= 0.8 on induction media (IM) supplemented with 200 μM of acetosyringone (AS) at 24°C for 48 hours, as described in the protocol for the AMT of *Colletotrichum *(Tsuji et al. 2003). Because the transformants were produced using, at most, 1 × 10^4 ^of *A. aculeatus *conidia (data not shown), we further assessed the AMT conditions on IM plates with regard to the ratio of *A. tumefaciens *and *A. aculeatus *cells, the duration of co-cultivation, and the *A. aculeatus *starting material. Various concentrations of *A. tumefaciens *cells, at OD_660 _= 0.2-0.8, were co-cultivated with 1 × 10^4 ^of *A. aculeatus *conidia at 24°C for 24, 48, and 72 hours. The results in Table 3 demonstrate that the transformation frequency increased in relation to the co-cultivation time and bacterial dosage, although prolonged co-cultivation periods (at 72 hours) and co-cultivation using a high concentration of *A. tumefaciens *(OD_660 _= 1.0) tended to yield transformants with severe growth defects such as impaired hyphal elongation and conidiation. We consequently obtained a maximum transformation frequency of 30 transformants per 1 × 10^4 ^conidia, on average, when 1 × 10^4 ^conidia of *A. aculeatus *were mixed with 1 × 10^8 ^bacterial cells (OD_660 _= 0.8) and co-cultivated for 48 hours on IM plates. Protoplasts and conidia were transformed with equal efficiency by *A. tumefaciens *(data not shown), which enabled us to omit the intricate handling for protoplast preparation. The relatively large standard deviation in these and later experiments presumably reflects the general nature of the transformation in *Aspergillus*.

**Table 3:** Optimization of ratios of fungal conidia to bacterial cells and co-cultivation periods on IM plates

Number of conidia	OD_660 _of *Agrobacterium* culture	Ratio of conidia: *Agrobacterium*	Mean of transformants ± SD/10^4^ conidia		
			
			24 h	48 h	72 h
1 × 10^4^	0.2	1:2.5 × 10^3^	n.d.^b^	8 ± 7 (n = 4)	n.d.
1 × 10^4^	0.4	1:5 × 10^3^	n.d.	7 ± 6 (n = 6)	n.d.
1 × 10^4^	0.6	1:7.5 × 10^3^	n.d.	10 ± 7 (n = 4)	n.d.
1 × 10^4^	0.8	1:10^4^	1 ± 1 (n^a ^= 2)	30 ± 28 (n = 12)	34 ± 27 (n = 2)
1 × 10^4^	1.0	1:1.25 × 10^3^	n.d.	62 ± 20 (n = 2)	n.d.

^a ^n, number of independent experiments.

^b^n.d., not done.

One rationale for optimizing AMT conditions for *A. aculeatus *was to allow insertional mutagenesis by T-DNA insertion. To help reduce the labor requirement of the numerous media preparations or transfer of many transformants from IM to SM plates, we investigated ways in which more transformants could be obtained on an SM plate by increasing the total amount of mixed *A. tumefaciens *(OD_660 _= 0.8) and conidia spread onto an IM plate while holding the ratio of conidia to *A. tumefaciens *cells at the optimum value (1:10^4^). Unexpectedly, increasing the amount of this mixture did not increase the number of transformants per plate in a dose-dependent manner because the transformation frequency was reduced (Table 4). This result suggests that critical parameters for efficient AMT include not only the ratio between bacterial cells and recipient cells, but also the density of their mixture during the infection.

**Table 4:** The effect of concentration of the fungal and bacterial cells on AMT

Number of conidia	Amount of Agrobacteria culture (ml)	Ratio of conidia: *Agrobacterium*	Mean of transformants ± SD/plate	Number of transformants /10^4 ^conidia
1 ×10^4^	0.1	1:10^4^	30 ± 28 (n^a ^= 12)	30 ± 28
2 ×10^4^	0.2	1:10^4^	52 ± 10 (n = 2)	26 ± 5
5 ×10^4^	0.5	1:10^4^	51 ± 13 (n = 2)	10 ± 2
1 ×10^5^	1	1:10^4^	39 ± 14 (n = 6)	3 ± 1
1 ×10^6^	10	1:10^4^	16 ± 9 (n = 3)	< 1

^a ^n, number of independent experiments.

### Optimization of AMT conditions of *A. aculeatus *in liquid IM

We presumed that the failure to increase the transformant yield by increasing the total number of conidia and bacterial cells per plate was the result of the inefficient infection of the fungus by *A. tumefaciens *on IM plates. Therefore, we expected that transformants could be obtained from a high density of infected germlings on SM plates after *A. tumefaciens *cells had successfully infected the fungus at the best ratio and concentration, followed by filtration to concentrate the infected germlings and subsequent transfer to SM plates. Furthermore, it has been reported that the co-cultivation of *B. lamprospora *in liquid IM facilitates the transmission of the foreign DNA when compared with cultivation on the surface of solid IM (Nyilasi et al. 2008). We next optimized the AMT conditions using liquid IM. As shown in Table 5, we obtained 217 transformants, on average, when 1 × 10^7 ^conidia of *A. aculeatus *were mixed with 5 × 10^8 ^bacterial cells and co-cultivated in 100 ml of liquid IM including 200 μM of AS for 48 hours with shaking at 120 rpm. Among the concentrations of AS investigated (0, 50, 100, 200, and 400 μM), 200 μM was selected for subsequent trials because this concentration yielded the most transformants. Co-cultivation for 60 hours yielded more transformants than at 48 hours; however, more transformants with growth defects tended to emerge on the SM plates. We again investigated the effects of a higher concentration of *A. tumefaciens *cells and conidia in liquid IM with the same ratio of *A. tumefaciens *cells to conidia (50:1), but the number of transformants did not increase in a dose-dependent manner. This result may have been caused by an insufficient supply of AS for the *A. tumefaciens *cells to express the virulence genes because the concentration of AS was held at 200 μM. However, keeping the effective concentration of AS at the optimal level in relation to the amount of *A. tumefaciens *cells did not increase the number of transformants obtained (data not shown). Although the transformation frequency in liquid IM showed a 100-fold reduction compared with the solid IM, this transformation method is suitable for random insertional mutagenesis because fewer SM plates are required for the transfer of transformants from IM to SM plates. Therefore, we propose that performing AMT using liquid IM is a practical means for high-throughput insertional mutagenesis.

**Table 5:** Optimization of AMT conditions in 100 ml of liquid IM

Number of conidia	Number of *Agrobacterium* cells	Ratio of conidia: *Agrobacterium*	Mean of transformants ± SD/10^7^ conidia/100 ml IM (n^a ^≥3)				
			
			24 h	36 h	48 h	60 h	72 h
1 ×10^7^	5 ×10^6^	1: 0.5	1 ± 4	16 ± 15	40 ± 27	41 ± 23	25 ± 16
1 ×10^7^	5 ×10^7^	1: 5	16 ± 27	60 ± 29	92 ± 88	55 ± 16	41 ± 26
1 ×10^7^	5 ×10^8^	1: 50	23 ± 20	132 ± 82	217 ± 141	292 ± 211	148 ± 101
1 ×10^7^	5 ×10^9^	1: 500	5 ± 8	3 ± 5	11 ± 12	10 ± 8	11 ± 14

^a ^n, number of independent experiments.

### Optimizing AMT conditions for different isolates

The transformation frequency tends to vary among different isolates of the same fungal species when AMT is performed using a method optimized for the standard strain (Roberts et al. 2003; Sullivan et al. 2002). The transformation frequency is also affected by slight differences in transformation conditions or the physiological state of the recipient cells (Michielse et al. 2005a). To investigate the AMT frequencies of different *A. aculeatus *isolates, we first compared the AMT frequencies of the *A. aculeatus *wild-type and a uridine auxotroph, the *pyrG *mutant. Because uridine must be added to liquid IM and SM plates to grow the *pyrG *mutant, we first assessed the effect of uridine addition on the AMT of the *A. aculeatus *wild-type (Table 6). In the wild-type, although the addition of 0.2% uridine to the liquid IM did not affect the number of transformants per 10^7 ^conidia per 100 ml IM, the addition of 0.01% uridine, which was the minimum concentration for the growth of the *A. aculeatus pyrG *mutant on MM plates, reduced the number of transformants by half in all trials except for the 24 h co-cultivation period (Table 6). Using conidia from the *pyrG *mutant as a starting material, the maximum number of transformants (135 ± 155) per 10^7 ^conidia per 100 ml IM was obtained at 60 h of co-cultivation with 0.2% uridine. The reduction of the transformation frequency and the long duration of the co-cultivation compared with the wild-type may be related to the reduced germination rate of the recipient conidia because the *pyrG *mutant never germinates or forms transformants in AMT without the addition of uridine to liquid IM (data not shown). Taking these data into account, we presumed that the germination of conidia and the physiological conditions of the recipient cells were critical for T-DNA uptake in *A. aculeatus*. Thus, we next investigated the AMT conditions using germinated conidia from the *pyrG *mutant that were pregrown for 24 h. Co-cultivation for 36 h with 0.01% uridine produced 122 transformants per 10^7 ^conidia per 100 ml of IM on average, which was 2- to 5-fold more than the amount obtained by the AMT method optimized for the wild-type strain; i.e. 48-hours co-cultivation with 0.01% uridine (24 ± 33 transformants) or 0.2% uridine added (58 ± 95 transformants). Our data demonstrate that the optimization of AMT for each isolate is necessary to establish efficient AMT methods.

**Table 6:** Optimization of transformation conditions for *A. aculeatus pyrG *mutant

Strain	Germination time (h)	Uridine Concentration in IM	Co-cultivation time and Mean of transformants ± SD /10^7 ^conidia/100 ml IM				
			
			24 h	36 h	48 h	60 h	72 h
WT	0	-	23 ± 20	132 ± 82	217 ± 141	292 ± 211	148 ± 101
	0	0.01%	81 ± 72	73 ± 36	116 ± 52	147 ± 64	82 ± 45
	0	0.2%	10 ± 12	193 ± 98	174 ± 106	187 ± 130	131 ± 118

*pyrG^-^*	0	0.01%	0	50 ± 38	24 ± 33	15 ± 12^a^	16 ± 13
	0	0.2%	0	66 ± 71	58 ± 95	135 ± 155^a^	33 ± 54
	24	0.01%	91 ± 66	122 ± 62	50 ± 49	140 ± 82^a^	50 ± 60
	24	0.2%	38 ± 24	26 ± 10	23 ± 11	2 ± 5^a^	12 ± 18

WT, the *A. aculeatus *wild-type strain; n = 3 except for a (n = 2)

### Mitotic stability of the integrated T-DNA

The fates of the T-DNA in the genomes of the transformants were assessed by Southern blot analyses using genomic DNA isolated from 120 randomly selected transformants and a DNA probe that hybridizes to the *hph *gene. These results revealed that the T-DNA integrated into the genomes of all transformants at random because DNA bands of various sizes were hybridized. The mitotic stability of the integrated DNA in 9 randomly selected transformants was examined after 5 rounds of mitosis on MM without hygromycin B, followed by culture on MM including hygromycin B. The average and standard deviation of the ratio of the number of colonies formed on MM with hygromycin B to that on MM without hygromycin B was 1.1 ± 0.13. The colony morphology also remained unchanged during culture. These data indicate that the integrated T-DNA is stably maintained in the recipient genome.

### Integration mode of the T-DNA into *A. aculeatus *genomic DNA

The effect of the co-cultivation conditions on the T-DNA integration pattern was investigated by Southern blot analyses. The recipient genomic DNA was isolated from transformants obtained under the following co-cultivation conditions: the ratio of bacterial cells to target conidia was 5 × 10^3 ^or 1 × 10^4 ^on solid IM (Table 3, 48 h) and 50 in liquid IM (Table 5, 48 h). Twenty, sixty, and forty transformants obtained under each set of conditions were randomly selected and analyzed. The overall frequencies of the single-locus integration of the T-DNA were 95%, 90%, and 90%, respectively (Table 7). Integration events predominantly occurred at a single locus under all the tested conditions, whereas further itemization of the integration pattern revealed differences. When the co-cultivation was performed on solid IM for 48 h at the ratio of *A. tumefaciens *to target conidia of 5 × 10^3^, and which yielded 7 transformants per 1 × 10^4 ^conidia (Table 3), on average, the T-DNA predominantly integrated into the recipient genome as a single copy (50%). Increasing the ratio of *A. tumefaciens *to target conidia to 1 × 10^4 ^improved the transformation frequency to 30 transformants per 1 × 10^4 ^conidia (Table 3, 48 h) on average; the single-copy integration of the T-DNA decreased to 30%, but the frequency of T-DNA integration into a single locus with the vector backbone typically increased to 55%. When the co-cultivation was performed in liquid IM at the ratio of *A. tumefaciens *to target conidia of 50, the yield was 217 transformants per 1 × 10^7 ^conidia, and single-copy integration of the T-DNA was predominant (40%). The T-DNA integration with the vector backbone was also relatively low. The AMT method optimized for liquid IM resulted in more transformants harboring the T-DNA integrated into a single locus without the vector backbone. Thus, we concluded that co-cultivation in liquid IM was suitable for the AMT of *A. aculeatus*.

**Table 7:** T-DNA integration patterns observed in transformants obtained under the indicated co-cultivation conditions

Integration pattern	Number of transformants		
	
	1:5000 on plate^a^	1:10^4 ^on plate^a^	1:50 in liquid^a^
1 copy	10 (50.0%)	18 (30.0%)	16 (40.0%)
1 copy + vector backbone	3 (15.0%)	14 (23.3%)	5 (12.5%)

multiple copies at single locus	5 (25.0%)	3 (5.0%)	8 (20.0%)
multiple copies at single locus + vector backbone	1 (5.0%)	19 (31.7%)	7 (17.5%)

multiple copies at different loci	1 (5.0%)	1 (1.7%)	1 (2.5%)
multiple copies at different loci + vector backbone	0 (0%)	5 (8.3%)	3 (7.5%)

Number of transformants analyzed	20 (100%)	60 (100%)	40 (100%)

a, the ratio of *A. aculeatus *conidia to *A. tumefaciens *cells.

### Recovery of flanking sequences

To obtain the DNA sequences flanking the T-DNA inserts in the recipient genome, we adopted TAIL-PCR and inverse PCR using genomic DNA isolated from randomly selected transformants. We first performed TAIL-PCR, which produced readable segments of the recipient genome adjacent to both sides of the T-DNA from all transformants (13/13 transformants) harboring the T-DNA as a single copy. However, when the T-DNA existed in the recipient genome with the vector backbone or as concatemeric bands with or without the vector backbone at a single locus, segments derived from the vector or the T-DNA tended to be amplified rather than the recipient genome. Therefore, the DNA sequences flanking the T-DNA inserts could only be identified on one side in 5 of 13 transformants. The T-DNA integration with a vector backbone made it difficult to rescue the flanks of the T-DNA border. To rescue the recipient genome flanking the T-DNA irrespective of its integration mode, we performed inverse PCR or TAIL-PCR on the recipient genome digested with restriction enzymes, as described in the Materials and Methods section. We considered that the trimming of a vector backbone or concatemeric T-DNA fragments attached to the recipient genomic DNA would increase the rescue rate for either side flanking the T-DNA. Indeed, the flanks were obtained in 5 out of 6 transformants by TAIL-PCR and 5 out of 5 transformants by inverse PCR using recipient genomic DNA digested with restriction enzymes. However, it remains challenging to rescue the flanks if the vector backbone is attached to both sides of the T-DNA or if the selected restriction sites do not exist near the T-DNA integration locus. In this case, we confirmed that the far side of the recipient genome was effectively rescued by TAIL-PCR using circular DNA produced by digestion with a restriction enzyme followed by ligation. Furthermore, we were able to identify T-DNA flanking sequences even though the T-DNA fragments were integrated into different loci, although only in one transformant. Our data indicate that the tagged genes in almost all of the transformants could be recovered by a combination of TAIL-PCR and inverse PCR, which satisfies the requirement for a successful gene tagging protocol.

**Figure 1:**
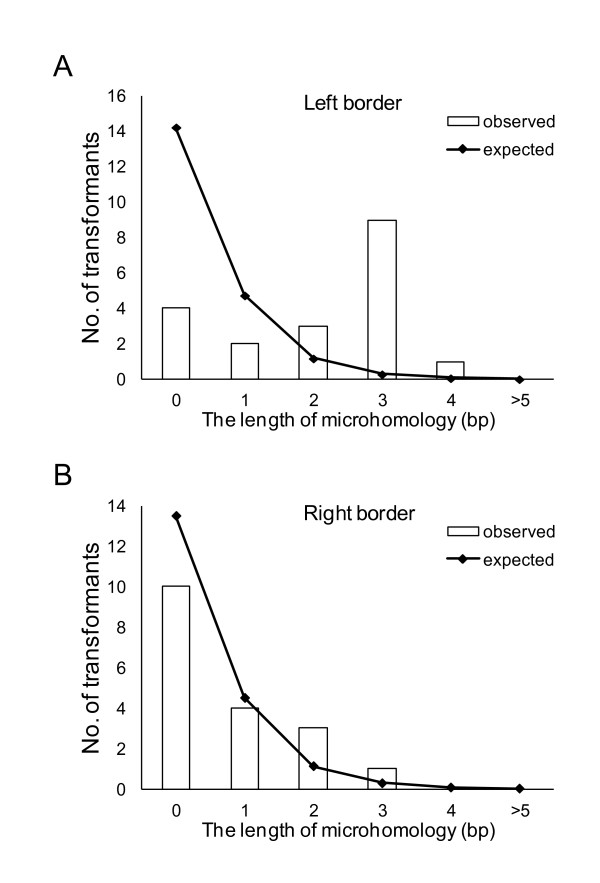
**Distribution of the regions with microhomology between the host genome and the left-border (A) and right-border (B) sequences**. The open bars show the distributions of T-DNA possessing each microhomologous region, and the solid lines show the expected length of microhomology.

Based on the above sequence analyses, we investigated how the T-DNA was inserted into the recipient genome without a large deletion of the T-DNA bordering sequence or the recipient genome sequence. Except in cases where the recipient genome was obtained with a short vector sequence, truncation of the T-DNA termini occurred in 89.5% (17/19) and 15.8% (3/19) of the border sequences on the left and right borders, respectively. Although truncations occurred with high frequency at the left terminus, the length of the truncation at the left T-DNA terminus was 8 bp on average and 42 bp at the longest. As shown in Figure 1A and B, the fungal DNA at the integration site had obvious microcomplimentarities with the left terminus of the T-DNA but not with the right terminus. This requirement of short stretches of homology at crossover points may have led to deletions at the left terminal integration sites.

**Figure 2:**
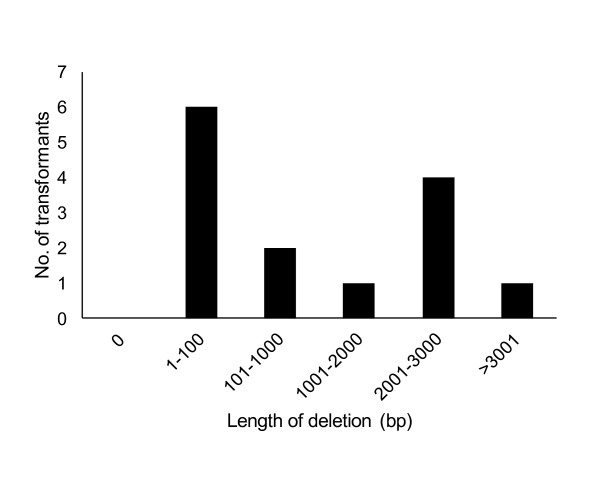
**A frequency distribution for different size classes of recipient genome deletions among 13 T-DNA integration sites for which the sequences of both junctions were determined**.

A comparison of both sides of the flanking sequence tags adjacent to the T-DNA with the draft genome sequence of *A. aculeatus *revealed that deletion of the recipient genome occurred in all 14 transformants analyzed, and the average length of the deletions was 1,393 bp. As shown in Figure 2, the predominance of deletions, 6 out of 14 (42.9%), was shorter than 100 bp. Deletions longer than 2,001 bp, including the longest deletion of 6,913 bp, occurred in 5 transformants (35.7%). Such deletions are acceptable for functional genomic analyses in *A. aculeatus*.

### Isolation and characterization of albino mutants

**Figure 3:**
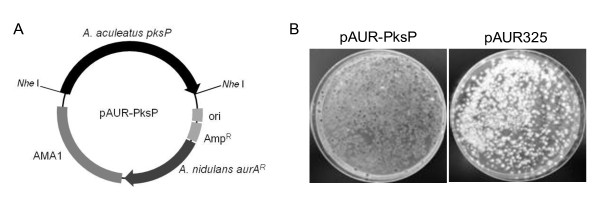
**Complementation of albino mutants**. (A) Diagram of the transformation vector for the *A. aculeatus alb1 *and *alb2 *mutants. (B) Pigmented colonies formed in transformants of *alb1 *with pAUR-pksP (left), but transformants with pAUR325 remained albino (right).

During the process of establishing our AMT protocols, 2 albino mutants, *A. aculeatus alb1 *and *alb2*, which formed colorless conidia, emerged on selective media from among approximately 11,000 transformants. Using these mutants, we assessed whether or not the established AMT method was applicable for random insertional mutagenesis. We first indentified genes disrupted by the T-DNA insertion in the *alb1 *mutant. A Southern blot analysis revealed that the T-DNA was inserted into a single locus in the *alb1 *mutant, so we performed TAIL-PCR to identify the T-DNA flanking sequences. A sequence analysis of the amplified flanks revealed that the T-DNA was inserted at 70 bp upstream of the polyketide synthase gene (the *pksP *gene (*AapksP*), Accession No. AB576490) and caused a 1,002-bp deletion in the recipient genome, which resulted in the deletion of a putative TATA box on the *pksP *promoter. *AapksP *was the only predicted gene near the T-DNA integration locus; it had 69.5% identity to the *wA *gene of *A. nidulans *(Accession no. Q03149) and 68.7% identity to the *pksP *gene of *A. fumigatus *(Accession no. EDP55264), which are involved in melanin biosynthesis and conidial pigmentation. To confirm that the deletion of the *Aapksp *locus resulted in the formation of the albino mutant, complementation tests were performed (Figure 3A). Transformation of the *alb1 *mutant with pAUR-PksP yielded transformants with black conidia, whereas all transformants of *alb1 *with pAUR325 remained albino (Figure 3B). Furthermore, the albino phenotype of the *alb2 *mutant was also complemented by transformation with pAUR-PksP (data not shown). Therefore, the mutation point resulting in the albino mutant corresponded to the locus for which the sequence was obtained as the T-DNA flank, thus demonstrating that AMT is a useful toolkit for gene tagging.

## Discussion

The results presented here demonstrate that the developed AMT method is applicable for high-throughput insertional mutagenesis in *A. aculeatus*. This method was developed by optimizing parameters that affect the AMT frequencies such as AS concentration, the ratio of *A. tumefaciens cells *to *A. aculeatus *cells, co-cultivation conditions, and starting materials (Michielse et al. 2008). Using the AMT method optimized for *A. aculeatus *wild-type, 30 transformants per 10^4 ^conidia were formed, on average, when using solid IM for co-cultivation. The transformation frequency on solid IM was relatively higher than that for other fungi, e.g., 150-300 transformants per 10^6 ^recipients in *C. lagenarium*, 200 transformants per 10^6 ^recipients in *A. awamori*, 5 transformants per 10^7 ^recipients in *A. niger*, and 50 transformants per 10^5 ^recipients in *N. crassa *(de Groot et al. 1998; Tsuji et al. 2003). *A. tumefaciens *C58C1 and a binary vector, pBIG2RHPH2, seem to be appropriate for the AMT of *A. aculeatus *because the transformation frequency is influenced by differences in the binary vector or bacterial strain used (Mullins et al. 2001).

Another aspect that must be considered to establish efficient AMT is the integration mode of the T-DNA into the recipient genome. In *F. oxysporum, A. awamori*, and *C. lagenarium*, single-copy integration events are predominantly observed (de Groot et al. 1998; Mullins et al. 2001; Tsuji et al. 2003). However, the fate of the T-DNA in *A. aculeatus *depends on the AMT method. A high density of *A. tumefaciens *increased the transformation frequency; however, undesirable integrations of the T-DNA such as multiple-copy integrations at a single locus, with or without the vector backbone, or integration at multiple loci also increased in *A. aculeatus*, as previously reported in *Blastomyces dermatitidis *(Sullivan et al. 2002) and in *Suillus bovinus *(Hanif et al. 2002). In contrast, a low density of *A. tumefaciens *yielded fewer transformants; however, the T-DNA predominantly existed as a single copy in the recipient genome. Although we expected to obtain more transformants by co-cultivating *A. tumefaciens *cells and fungal conidia in high quantities on solid or liquid IM, this technique did not yield more transformants. There is a limit to the concentration of bacterial cells and fungal conidia for efficient AMT that is likely related to the physiological conditions of *A. tumefaciens *and the recipient fungal cells (Meyer et al. 2003). The best AMT method should increase the number of transformants harboring the T-DNA as a single copy, which is a criterion with apparently opposing contributing factors. Thus, we hereby propose that AMT using liquid IM satisfies this criterion for random insertional mutagenesis.

The transformation frequency for the *A. aculeatus *uridine auxotroph tended to be lower than that of the wild type with the same AMT method (Table 6). When acetamide utilization, uridine prototrophy, phleomycin resistance, or hygromycin resistance was used to select transformants in *A. awamori*, average transformation frequencies of 0.2, 40, 80, and 200 transformants per 10^6 ^conidiospores were obtained (Michielse et al. 2008). The transformation frequency apparently varies depending on the genetic background of the recipient and the transformation conditions (Covert et al. 2001; de Groot et al. 1998; Fitzgerald et al. 2003; Michielse et al. 2004; Sullivan et al. 2002). However, the transformation frequency among different isolates has been assessed by using the same AMT method. Our data show an improvement of transformation frequency by optimizing AMT conditions according to the recipient cells, and which supports that AMT means can be applicable to transform variety types of the recipient cells.

During the optimization of AMT conditions, 2 albino mutants, the *Aapksp *disruptants, were discovered among 11,000 transformants. To isolate one albino mutant with a 95% probability out of 5,500 transformants from the approximately 35-Mb genome of *A. aculeatus*, it was calculated that the T-DNA must disrupt gene the function in a 19-kb region around the T-DNA integration locus (Krysan et al. 1999). The 1,002-bp deletion at the *AapksP *locus in the *alb1 *mutant was relatively larger than those reported for *M. oryzae *and *Arabidopsis*, in which the majority of deletions ranged from 1 to 35 bp and 11 to 100 bp, respectively (Brunaud et al 2002; Choi et al. 2007; Forsbach et al. 2003). Furthermore, the coding region of *AapksP *is relatively large at 6,645 bp; however, it is not large enough to increase the probability of isolating two albino mutants from 11,000 transformants. Although the T-DNA integration was initially thought to be random in *Arabidopsis *(Azpiroz-Leehan and Feldmann, 1997) and yeast (Bundock et al. 2002), the randomness of the integration has been controversial given the accumulated data indicating the nonrandom nature of T-DNA insertion. In *Arabidopsis*, the T-DNA integration occurs between the recipient genome and microhomologous sequences of the T-DNA composed of 5-bp and 2-bp sequences on the left border and right border, respectively (Brunaud et al. 2002). Furthermore, obvious biases were reported for insertions in the 5'- and 3'-regulatory regions outside the coding regions for 500-bp regions and in introns rather than in exons in the rice genome (Chen et al. 2003), outside of the transcribed regions in *Arabidopsis *(Sessions et al. 2002), and in the intergenic region in *M. oryzae *(Choi et al. 2007). In *A. aculeatus*, microhomology was observed on the left border of the T-DNA; however, the other bias has not been identified thus far, although we are in the process of analyzing more transformants. A combination of T-DNA bias integration and a certain deletion length in the recipient genome can contribute to the facilitation of high-throughput insertional mutagenesis by AMT. We expect that the AMT method established here will contribute to functional genetic analyses in *A. aculeatus*. Moreover, we expect that the techniques described here can be applied to establish AMT techniques for other organisms.

## Competing interests

The authors declare that they have no competing interests.
